# Poly(ADP-ribose) polymerase 1 at the crossroad of metabolic stress and inflammation in aging

**DOI:** 10.18632/aging.100052

**Published:** 2009-05-20

**Authors:** Matthias Altmeyer, Michael O. Hottiger

**Affiliations:** ^1^ Institute of Veterinary Biochemistry and Molecular Biology, University of Zurich, Zurich, Switzerland; ^2^ Life Science Zurich Graduate School, Molecular Life Science Program, Zurich, Switzerland

**Keywords:** PARP-1, NAD+, ROS, NF-κB, inflammation, aging

## Abstract

Poly(ADP-ribose) polymerase 1 (PARP1) is a chromatin-associated nuclear protein, which functions as molecular stress sensor. Reactive oxygen species, responsible for the most plausible and currently acceptable global mechanism to explain the aging process, strongly activate the enzymatic activity of PARP1 and the formation of poly(ADP-ribose) (PAR) from NAD^+^. Consumption of NAD^+^ links PARP1 to energy metabolism and to a large number of NAD^+^-dependent enzymes, such as the sirtuins. As transcriptional cofactor for NF-κB-dependent gene expression, PARP1 is also connected to the immune response, which is implicated in almost all age-related or associated diseases. Accordingly, numerous experimental studies have demonstrated the beneficial effects of PARP inhibition for several age-related diseases. This review summarizes recent findings on PARP1 and puts them in the context of metabolic stress and inflammation in aging.

## Introduction

Aging is a multi-factorial process defined as time-dependent general decline in physiological function, which is associated with a progressively increasing risk of frailty, morbidity and mortality [1,2]. The effect of aging is mainly observed in modern human societies and in animals under laboratory conditions [3]. The dramatic increase in mean human life span and life expectancy, coupled to a significant reduction in early mortality caused by the reduced occurrence of infections during the past two centuries, has led to an enormous increase in the number of elderly people in modern societies [4,5]. This demographic phenomenon has been paralleled by an epidemic of chronic diseases associated with advanced age, most of which have complex etiology and underlying pathogenic mechanisms [6]. Intensive efforts have been made over the last decades to identify single key players involved in age-related diseases. Poly(ADP-ribose) polymerase 1 (PARP1) is a chromatin-associated nuclear protein which functions as stress sensor and as such is involved in the cellular responses to a variety of age-related stress signals.

### Poly(ADP-ribose) polymerase 1 as molecular stress sensor

PARP1 is an abundant nuclear chromatin-associated multifunctional enzyme found in most eukaryotes apart from yeast [7]. PARP1 has been initially thought to be the only existing enzyme with poly(ADP-ribosyl)ation activity in mammalian cells. However, five additional *Parp*-like genes encoding *"bona fide"* PARP enzymes have been identified in recent years, indicating that PARP1 belongs to a family of "*bona fide"* PARP enzymes [8]. The basal enzymatic activity of PARP1 is very low, but is stimulated dramatically under conditions of cellular stress [9,10]. Activation of PARP1 results in the synthesis of poly(ADP-ribose) (PAR) from nicotinamide adenine dinucleotide (NAD^+^) and in the release of nicotinamide as reaction by-product [7,8]. Following PARP1 activation, intracellular PAR levels can rise 10-500-fold [11-13]. Despite intensive research on the cellular functions of PARP1, the molecular mechanism of PAR formation has not been comprehensively understood. Up to now, two different modes of PARP1 activation have been described, one dependent on DNA damage and one dependent on post-translational protein modifications (see below).

PAR is a heterogeneous linear or branched homo-polymer of repeating ADP-ribose units linked by glycosidic ribose-ribose bonds [7,9,14]. Most free or protein-associated PAR molecules are rapidly degraded *in vivo* [15]. This rapid turnover strongly suggests that PAR levels are tightly regulated under physiological stress conditions and that degradation of the polymer starts immediately upon initiation of PAR synthesis. To date two enzymes, poly(ADP-ribose) glycohydrolase (PARG) and ADP-ribosyl protein lyase, have been described to be involved in PAR catabolism [16,17]. While PARG possesses both exo- and endoglycosidic activities, the lyase has been described to cleave the bond between proteins and mono(ADP-ribose). The attachment of negatively charged PAR onto proteins is transient but can be very extensive *in vivo*, as polymer chains can reach more than 400 units on protein acceptors [7]. PAR formation has been implicated in a variety of cellular processes, such as maintenance of genomic stability, transcriptional regulation, energy metabolism and cell death [7]. The physiological consequences of this post-translational modification on the molecular level, however, are not yet completely understood. It has been proposed that PAR may have a dual role in modulating cell survival and cell death [9,18,19]. Low to moderate levels of PAR may be beneficial for important cellular functions, whereas extensive PAR formation can be detrimental and lead to various forms of cell death. More than a decade ago, PARP1 activity was linked to the aging process, as poly(ADP-ribosyl)ation capacity was shown to correlate with species-specific longevity [20,21].

Most proteins associated with PAR are nuclear DNA-binding proteins, including PARP family members and histones [7,22,23]. PARP1 is the main acceptor for poly(ADP-ribosyl)ation *in vivo* and auto-modification of PARP1 abolishes its affinity for NAD^+^ and DNA [24,25]. A similar effect has been postulated for histones/nucleosomes. PAR polymers could function to alter chromatin conformation through covalent or non-covalent interactions with histone tails and via displacement of histones from DNA, thus regulating the accessibility of the genetic material. It was suggested that PAR might either directly participate in chromatin remodelling processes or indirectly coordinate them through recruitment and regulation of specific chromatin remodelling proteins [7,22]. Moreover, PAR is recognized and bound by macrodomain containing histone variants [26].

Over 20 years ago, Nathan Berger was the first to suggest that cellular stress (e.g. oxidative damage) causes over-activation of PARP1 and subsequent NAD^+^ depletion [27,28]. In an attempt to restore the NAD^+^ pools, NAD^+^ is resynthesized with a consumption of 2-4 molecules of ATP per molecule of NAD^+^. As a consequence, cellular ATP levels become depleted, leading to subsequent energy failure, which results in cellular dysfunction and eventually in necrotic cell death [27,28]. Pharmacological inhibition of the enzymatic activity of PARP or the complete absence of PARP1 was shown to significantly improve cellular energetic status and cell viability after exposure to necrosis-inducing agents [29-31]. The contribution of poly(ADP-ribosyl)ation reactions to necrotic cell death seems to be dependent on the cell type and the cellular metabolic status [7,32,33].

Interestingly, genetic studies using *Parp1 *knockout mice provided preliminary evidence that energy depletion alone might not be sufficient to mediate poly(ADP-ribosyl)ation-dependent cell death [34]. A second model has been proposed to explain how PARP1 regulates cell death. This model suggests that over-activation of PARP1 induces translocation of apoptosis-inducing factor (AIF) from the mitochondria to the nucleus, causing DNA condensation and fragmentation, and subsequent cell death [35].

Together, PARP1 can be regarded as molecular stress sensor with many physiological cellular functions. Over-activation of PARP1 results in the generation of large amounts of PAR. Subsequently, cellular NAD^+^ pools are depleted and AIF is released from the mitochondria to trigger cell death. Importantly, these PARP1-dependent cellular suicide mechanisms have been implicated in the pathomechanisms of neuro-degenerative disorders, cardiovascular dysfunction and various other forms of inflammation [36].

### Activation of PARP1 by reactive oxygen species (ROS)

A unified theory explaining the pathogenesis of diverse degenerative conditions in different organs (including Alzheimer's, Parkinson's and other neurodegenerative disorders, rheumatoid arthritis, atherosclerosis and other cardiovascular diseases, diabetes) has been proposed to explain how the single physiological process of aging may lead to diverse pathological states [37]. This oxidative stress theory of aging (or free radical theory of aging), initially proposed by Harman in 1956, provides the most plausible and currently acceptable global mechanism to explain the aging process [38]. The theory postulates that aging is, in the absence of other risk factors (e.g. infections, smoking, hypercholesterolemia), the net consequence of free radical-induced damage and the inability to counter-balance these changes by anti-oxidative defenses. An increase in intracellular ROS levels through hydrogen peroxide treatment of cells or through the inhibition of ROS scavenging enzymes, such as superoxide dismutase (SOD1), causes premature senescence and can shorten cellular life span [39-45]. Mitochondria are the main producers of cellular ROS under normal conditions, as approximately 1-2% of the oxygen molecules consumed during respiration are converted into highly reactive superoxide anions [46]. Besides aerobic metabolism in mitochondria, β-oxidation in peroxisomes and certain enzymes can produce ROS. Intracellular ROS can damage cellular components through oxidation of macromolecules such as nucleic acids, proteins and lipids [47]. Moreover, an overproduction of ROS leads to rapid generation of peroxinitrite from nitric oxide and superoxide, causing an imbalance in nitric oxide signaling [48].

Since the oxidative stress theory was first proposed, a considerable body of evidence has been published corroborating the idea that increased production of ROS underlies cellular dysfunction in various organ systems of aged humans and laboratory animals [49]. Interestingly, the enzymatic activity of PARP1 can be strongly activated by treatment of cells with ROS such as hydrogen peroxide [8]. Earlier studies described that PARP1 binds to oxidative damage-induced strand breaks within the DNA via two zinc finger motifs and thereby becomes activated [9]. More recently, several studies suggested that PARP1 activity is also regulated in a DNA-independent manner. A proteomic investigation uncovered many ERK1/2-induced phosphorylation sites in PARP1, which are located within important functional domains, consistent with regulatory roles *in vivo* [50,51]. Furthermore, DNA-independent PARP1 activation can be triggered by the direct interaction of PARP1 with phosphorylated ERK-2 without PARP1 being phosphorylated itself [52].  In addition, PARP1 can be activated by elevated levels of extracellular glucose, Ca^2+^ and angiotensin II, and allosteric regulation of auto-poly(ADP-ribosyl)ation by Mg^2+^, Ca^2+^, polyamines, ATP and the histones H1 and H3 has been reported [53]. Whether ROS-mediated activation of PARP1 is due to ROS-generated DNA damage or also based on other ROS-induced cellular (signaling) mechanisms awaits further investigations.

**PARP1 is linked to energy metabolism through NAD^+^**

NAD^+^ biosynthesis has become of considerable interest due to the important signaling functions of pyridine nucleotides. In mammals, niacin (collectively designating nicotinamide and nicotinic acid) and the essential amino acid tryptophan are precursors of NAD^+ ^biosynthesis [12,54]. The formation of dinucleotides from ATP and the mononucleotide of niacin constitute the most critical step in NAD^+^generation, which is catalyzed by NMN/NaMN adenylyltransferases (NMNATs) [13,55]. Since PARP1 uses NAD^+^ as substrate to synthesize PAR, PARP1 decisively depends on the amount of NAD^+^ available and may act as energy sensor in the nucleus. Both constitutive and activated levels of PAR have been suggested to be strictly dependent on the concentration of NAD^+^ in cells [15,56,57]. Importantly, the nuclear concentration of NAD^+ ^can be modulated by NMNAT-1 and a recent study revealed that NMNAT-1 is able to interact with and stimulate PARP1 [58]. It is thus tempting to speculate that PARP1 activation is supported by the localized action of NMNAT-1. Depending on the level of PARP1 activity, the cellular NAD^+^ concentration is concomitantly reduced. Therefore, PARP1 not only is a sensor of NAD^+^, but in turn also influences cellular energy levels.

Dietary restriction, also called calorie restriction, is defined as a life-long moderate (20-40%) reduction in caloric intake and has repeatedly been shown to extend the longevity of both invertebrates and vertebrates [59,60]. Reducing the caloric intake starting even at an old age has also been shown to increase the life span of flies and mice and is sufficient to reverse gene expression changes associated with aging [61-63]. Furthermore, dietary restriction in rodents delays the onset and reduces the severity of many age-related diseases, such as cardiovascular disease, diabetes, osteoporosis, cataracts, neurodegenerative disease and cancers [60]. Although it was initially expected that dietary restriction would reduce overall cellular energy levels byslowing down glycolysis and the tricarboxylic acid (TCA) cycle [59], this assumption has been challenged, since dietary restriction was shown to cause an increase in NAD^+^/NADH ratios in yeast cultures [64]. Whether this is also the case in mammalian cells remains to be determined. Along the same lines, the impact of dietary restriction on enzymes that depend on NAD^+^ (e.g. PARP1) is currently being investigated in multiple laboratories. Whether and how PARP1 activation differs in species with different maximal life span (and possibly also with different cellular NAD^+^ pools), however, remains an open question.

### Crosstalk between PARP1 and other NAD^+^-consuming enzymes

NAD^+^ is an essential cofactor regulating numerous cellular pathways and has recently been recognized as a substrate for a growing number of NAD^+^-dependent enzymes [11,13]. NAD^+^-dependent post-translational protein modifications are catalyzed by several enzyme families, including PARPs and the sirtuin family of NAD^+^-dependent class III histone deacetylases (SIRTs) [8,65,66]. SIRTs and the yeast homolog and founding member of the sirtuins, Sir2, are induced by dietary restriction and have been implicated in senescence and aging, although the exact mechanisms are not yet known [59,67]. Intriguingly, ADP-ribosylation by PARP1 could modulate the NAD^+^-dependent deacetylation of proteins by SIRTs via the NAD^+^/nicotinamide connection. The decline of NAD^+^ levels and the rise of nicotinamide upon PARP1 activation have immediate effects on other NAD^+^-consuming enzymes [57,68,69]. SIRTs require NAD^+ ^as substrate and are inhibited by low levels of nicotinamide [70]. Consequently, under conditions of cellular stress and PARP1 activation, the activity of SIRTs is downregulated.

PARPs and sirtuins may not only compete for the same substrate, but might also regulate each other more directly. For instance, PARP1 and SIRT1 interact at the protein level and SIRT1 might be regulated by PARP1-dependent trans-ADP-ribosylation [7]. Another link between PAR generation and acetylation/deacetylation reactions comes from the very recent identification of three lysine residues in the auto-modification domain of PARP1 as acceptor sites for auto-ADP-ribosylation [71]. The same lysines were previously identified as targets for acetylation by p300 and PCAF [72]. Remarkably, simple addition of PCAF reduced poly(ADP-ribosyl)ation of PARP1 (own unpublished observation), suggesting that the interaction domain of PARP1 with PCAF is overlapping with the ADP-ribose acceptor sites. We recently also published that acetylation of lysine residues interferes with ADP-ribosylation [73]. This finding points at an interesting crosstalk between acetylation of and ADP-ribosylation by PARP family members. It will certainly be interesting to further investigate the crosstalk between PARP1-dependent ADP-ribosylation  and acetylation/ deacetylation reactions. NAD^+^ levels can be expected to play an important role for the interplay between these two NAD^+^-dependent post-translational protein modifications. Whether the balance between and the tight regulation of poly(ADP-ribosyl)ation and NAD^+^-dependent deacetylation is altered during aging remains to be investigated. Furthermore, it will be important to identify additional NAD^+^-dependent enzymes involved in the aging process.

Emerging pathological evidence indicates that major chronic age-related diseases, such as atherosclerosis, arthritis, dementia, osteoporosis and cardiovascular disease, are inflammation-related [74]. A link between NAD^+^ metabolism and the regulation of an inflammatory response is suggested by the finding that nicotinamide phosphoribosyltransferase (NAMPT), one of the enzymes involved in NAD^+^ biosynthesis from nicotinamide, increases cellular NAD^+ ^levels in response to stress [75]. The expression of NAMPT is upregulated in activated lymphocytes [76]. Furthermore, NAMPT protein and/or mRNA levels were also found to be upregulated upon stimulation of immune cells both *in vivo* and *in vitro* [77,78], whereas a specific NAMPT inhibitor was found to inhibit cytokine production [79]. Notably, nicotinamide is known to inhibit the production of key inflammatory mediators [80-82], protects neurons against excitotoxicity [83,84], and blocks replicative senescence of primary cells [85]. Moreover, a recent study suggested that intracellular NAD^+^ levels regulate TNF-α protein synthesis in a SIRT6-dependent manner [86]. Both, SIRT1 and SIRT6 also regulate NF-κB signaling with effects on senescence and possibly aging [87,88].

Together, accumulating evidence suggests that cellular NAD^+^ biosynthesis and the NAD^+^-consuming reactions poly(ADP-ribosyl)ation and SIRT-dependent deacetylation are tightly interrelated and have functions in inflammation and age-related diseases.

### PARP1 is linked to age-related inflammation as transcriptional cofactor of NF-κB

A body of experimental and clinical evidence suggests that the immune system is implicated in almost all age-related or associated diseases [89,90]. There is a well-established connection between oxidative stress and the inflammatory immune response [37]. A prominent mechanism by which age-induced ROS modulate inflammation is by inducing the redox-sensitive transcription factor nuclear factor kappa B (NF-κB). This induction of NF-κB leads to the generation of pro-inflammatory mediators and a state of chronic inflammation [91,92]. NF-κB plays an important role in inflammatory phenotypic changes in various pathophysiological conditions [49]. In fact, NF-κB has a fundamental role in mediating all the classical attributes of inflammation - rubor, calor, dolor and tumor - by regulating transcriptional programs in tissues containing epithelial and stromal cells, vascular endothelial cells and hematopoietic cells [93]. During the last decade, it has been clearly demonstrated that excessive activation or inappropriate regulation of immune and inflammation cascades causes tissue and cellular damage, which can lead to cellular dysfunction and death [14]. Furthermore, it was suggested that chronic, low-grade inflammation is a possible converging process linking normal aging and the pathogenesis of age-related diseases [94]. This hypothesis is in accordance with the finding that constitutive activation of NF-κB, accompanied by elevated levels of inflammatory markers, is a ubiquitous phenomenon observed in various cell types in the aging phenotype [95].

In most unstimulated cells, NF-κB is sequestered in the cytoplasm as an inactive transcription factor complex by its physical association with one of several inhibitors of NF-κB (IκB) [96-100]. The key regulatory event in NF-κB induction is the phosphorylation of IκB proteins by the IκB kinase (IKK) complex, which leads to IκB protein ubiquitylation and subsequent degradation [101,102]. ROS have been reported to induce the activation of NIK/IKK and MAPK pathways that lead to the degradation of IκB and subsequent NF-κB-dependent gene expression [74,103]. Conversely, induction of NF-κB itself results in the generation of ROS via the expression of inducible nitric oxide synthase (iNOS), thus activating a feedback loop that amplifies the process of damage and deterioration in target cells and organs [37].

Global screens for age-specific gene regulation have been performed from many tissues in mice and humans [3]. These analyses have recently provided evidence that the NF-κB binding domain is the genetic regulatory motif most strongly associated with the aging process and thatNF-κB target genes show a strong increase in expression with age in human and mouse tissues as well as in stem cells [104-106]. Furthermore, NF-κB is implicated in age-dependent induction of cellular senescence in epithelial and hematopoietic progenitor cells [104,107]. Blockade of NF-κB in the skin of aged mice can reverse the global gene expression program and tissue characteristics to that of younger animals [108]. Moreover, Donato et al. reported lately that in vascular endothelial cells of aged human donors nuclear NF-κB levels increase, IκBα levels decrease and that the expression of proinflammatory cytokines, such as interleukin 6 (IL-6), tumor necrosis factor-α (TNF-α) and monocyte chemoattractant protein 1 (MCP-1) is reduced [109]. NF-κB activity was also increased in aged rat vessels and kidneys, but reduced in rats under calorie restriction [110,111].

Studies performed with *Parp1* knockout mice have identified various detrimental functions of PARP1 in inflammatory and neurodegenerative disorders. *Parp1* gene-disruption protected from tissue injury in various oxidative stress-related disease models ranging from stroke, (MPTP)-induced parkinsonism, myocardial infarction, streptozotocin-induced diabetes, lipopolysaccharide-induced septic shock, arthritis, to colitis and zymosan-induced multiple organ failure [7,73,112,113]. There are striking similarities between the expression pattern of PARP1 and the detrimental transcriptional activity of NF-κB. In most tissues and cell types associated with high PARP1 expression, dysregulated NF-κB activity seems to contribute to cellular dysfunction and necrotic cell death during inflammatory disorders [14]. The strongest indication for a direct role of PARP1 in NF-κB-dependent transcription was the impaired expression of NF-κB-dependent pro-inflammatory mediators in *Parp1 *knockout mice [113]. Moreover, the upregulation of several inflammatory response genes after treatment with inflammatory stimuli was drastically reduced in *Parp1* knockout mice [112,114-116]. Our group provided first evidence that PARP1 is required for specific NF-κB-dependent gene activation and can act as transcriptional coactivator of NF-κB *in vivo* [117]. PARP1 is required and sufficient for specific transcriptional activation of NF-κB in response to pro-inflammatory stimuli and cellular stress. Furthermore, Tulin and Spradling found that Drosophila mutants lacking normal PARP levels display immune defects similar to mice lacking the NF-κB subunit p50 [118]. These results imply that the role of PARP1 in NF-κB-dependent gene expression during immune responses has been conserved during evolution. Together, several lines of evidence suggest a model in which PARP1 functions as a promoter-specific cofactor for NF-κB-dependent gene expression [7,14].

### PARP as therapeutic target for age-associated diseases

During the last two decades of intensive research, over 50 potential PARP inhibitors were developed [119]. The involvement of PARP1 in cell death (both apoptosis and necrosis) and the capacity of PARP1 to promote the transcription of pro-inflammatory genes are particularly important for drug development. On the basis of structural information available for the catalytic domains of PARP1 and PARP2 co-crystallized with NAD^+^ or certain PARP inhibitors, it became clear that the majority of PARP inhibitors mimic the nicotinamide moiety of NAD^+^and bind to the donor site within the catalytic domain [120-122]. Although the physiological functions of PARPs and poly(ADP-ribosyl)ation is still under debate, numerous experimental studies during the last years have clearly demonstrated the beneficial effects of PARP inhibition from cell culture systems to pre-clinical animal models of acute and chronic inflammation [36,119]. For instance, Vaziri and colleagues observed an extension of cellular life span when PARP activity was inhibited [123]. In animal studies, PARP inhibition and/or PARP1 deficiency is effective in different age-related diseases [119]. The PARP inhibitor 5-AIQ has been demonstrated to attenuate the expression of P-selectin and intracellular adhesion molecule-1 (ICAM-1) as well as the recruitment of neutrophils and leukocytes into the injured lung [124,125]. Thus, application of inhibitors reduces the degree of acute inflammation and tissue damage associated with experimental lung injury. As ROS released from the recruited leukocytes cause an upregulation of adhesion molecules, treatment with PARP inhibitors contributes to the termination of this vicious cycle and inhibits the inflammatory process. Similar to the effects of pharmacological inhibitors, *Parp1* knockout mice were found to be resistant against zymosan-induced inflammation and multiple organ failure when compared with the response of wild-type animals [126].

In murine models of arthritis, inhibition of PARP with nicotinamide delayed the onset of the disease and reduced the progress of established collagen-induced arthritis [127]. 5-iodo-6-amino-1,2-benzopyrone and PJ34, two novel PARP inhibitors, were beneficial in a mouse model of collagen-induced arthritis by reducing both the incidence of arthritis and the severity of the disease [128,129]. Similarly, GPI 6150 was found to be highly effective in a rodent model of adjuvant-induced arthritis [130].

PARP activation also has a pathogenic role in hypertension, atherosclerosis and diabetic cardiovascular complications [119,131]. In these diseases, the function of the vascular endothelium is impaired, resulting in a reduced ability of the endothelial cells to produce nitric oxide and other cytoprotective mediators. This then sets the stage for many manifestations of cardiovascular disease. The oxidant-mediated endothelial cell injury is dependent on PARP1 and can be attenuated by pharmacological inhibitors or genetic PARP1 deficiency [115,132]. Furthermore, PARP inhibition improves aging-associated cardiac and endothelial dysfunction [133].

In general, the severity of many inflammatory diseases is suppressed by PARP inhibitors and the production of multiple pro-inflammatory mediators is downregulated [48]. The inhibition of PARP also reduces the formation of nitrotyrosine in inflamed tissues, an indicator of reactive nitrogen species. This finding was, at first, unexpected because PARP activation is perceived to occur downstream of the generation of oxidants and free radicals in various diseases. The mechanism is probably related to the fact that PARP inhibition reduces the infiltration of neutrophils into inflammatory sites [126]. This in turn reduces oxygen- and nitrogen-centered free-radical production. The basis for the regulation of neutrophil infiltration by PARP might be related to the reduced expression of adhesion molecules [134,135] and/or the preservation of endothelial integrity [115,132]. Alternatively, the reduction of nitrotyrosine could be explained by the finding that PARP1 is required for the expression of iNOS, the main producer of nitric oxide in inflamed tissues [116]. In summary, multiple studies suggest that a tight regulation of PARP activity is required to prevent a variety of age-related pathological conditions.

### Role of PARP1's enzymatic activity in NF-κB -dependent gene expression 

There is no consensus in the literature as to whether the modulation of NF-κB-mediated transcription by PARP1 is dependent on poly(ADP-ribosyl)ation or, alternatively, merely on the physical presence of PARP1 [14]. Genetic approaches provide strong evidence that poly(ADP-ribosyl)ation is not affecting the DNA binding activity of NF-κB and is not required for NF-κB-dependent gene expression [14,136]. Neither the enzymatic activity of PARP1 nor its binding to DNA was required for full activation of NF-κB in response to various stimuli *in vivo *when tested on transiently transfected reporter plasmids [137,138]*.* Consistently, the enzymatic activity of PARP1 was not required for full transcriptional activation of NF-κB in the presence of the histone acetyltransferase p300 [72]. At first glance this seems not to be compatible with reports describing that PARP inhibitors abolish mRNA expression of iNOS, IL-6 and TNF-α in cultured cells [139] or that PARP inhibitors reduce the expression of inflammatory mediators in mice [124,126,140]. However, this discrepancy might be explained in three ways: First, it should be noted that the currently available PARP inhibitors do not discriminate well between PARP1 and other PARP family members or even other NAD^+^-metabolizing enzymes, which are described to also play a role in inflammatory response pathways [139,141]. In *Parp1* knockout mice, PAR formation is indeed drastically reduced only in brain, pancreas, liver, small intestine, colon, and testis, whereas still moderate levels of residual poly(ADP-ribose) formation can be observed in the stomach, bladder, thymus, heart, lung, kidney and spleen [7]. This residual activity can most likely be attributed to PARP2, which has the greatest similarity to PARP1 among all PARP family members [8]. Interestingly, PARP2 is involved in T lymphocyte development and survival [142] and has been implicated in inflammatory immune responses [143,144]. A putative role of PARP2 in aging awaits further investigations. Second, based on recent reports, one cannot exclude the possibility that PARP-inhibitors might even affect non-NAD^+^-consuming targets such as AKT/PKB or MMPs [145]. Third, the enzymatic activity of PARP1 might be required for the transcriptional activity of transcription factors involved in inflammatory processes other than NF-κB. Several groups have shown that co-operative activities between transcription factors such as AP-1, STAT-1 or IRF-1 in the enhanceosomes of NF-κB dependent genes are required for full synergistic activation of target genes [146,147]. Considering these constraints of all currently available PARP inhibitors, the specific contribution of PARP1 enzymatic activity for age-related diseases, in which PARP inhibition has beneficial effects, needs to be evaluated very carefully.

**Figure 1.**
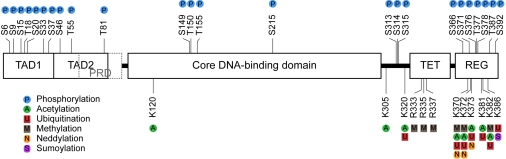
PARP1 at the crossroad of metabolic stress and inflammation in aging. PARP1 is activated by cellular stress, e.g. by oxidative damage due to increased levels of reactive oxygen species (ROS). As NAD^+^-dependent enzyme, PARP1 senses energy levels and crosstalks with other NAD^+^-consuming enzymes. Over-activation of PARP1 leads to energy depletion and cell death. On the other hand, PARP1 functions as cofactor for NF-κB-dependent transcription and is therefore implicated in many inflammatory processes. Both, PARP1-mediated metabolic stress and PARP1-regulated inflammation can lead to tissue degeneration underlying many age-related pathologies. See text for further details.

## Conclusions

Several publications in the past years indicate that the nuclear protein PARP1 represents a molecular link between energy metabolism and inflammation (Figure). As NAD^+^-consuming enzyme, PARP1 acts as nutrient or energy sensor, crosstalks with other NAD^+^-consuming enzymes (such as sirtuins) and modulates (as regulator of NF-κB-dependent transcription of cytokines) inflammatory responses. Thus, PARP1 seems to be an ideal candidate to integrate metabolic and inflammatory signals, which arise during the process of aging. As central integrator, PARP1 may mediate cellular stress response pathways and thereby participate in a multitude of age-related pathologies. PARP inhibition has proven beneficial in many cell culture and animal model systems of acute and chronic inflammation and age-related diseases. Clearly, addi-tional research will further improve our understanding of the functions of PARP1 and their implications in age-related diseases associated with metabolic stress and inflammation.

## References

[1] Murphy MP, Partridge L (2008). Toward a control theory analysis of aging. Annu Rev Biochem.

[2] Beneke S, Burkle A (2007). Poly(ADP-ribosyl)ation in mammalian ageing. Nucleic Acids Res.

[3] Kim SK (2007). Common aging pathways in worms, flies, mice and humans. J Exp Biol.

[4] Crimmins EM, Finch CE (2006). Infection, inflammation, height, and longevity. Proc Natl Acad Sci U S A.

[5] Finch CE, Crimmins EM (2004). Inflammatory exposure and historical changes in human life-spans. Science.

[6] Vasto S, Caruso C (2004). Immunity & Ageing: a new journal looking at ageing from an immunological point of view. Immun Ageing.

[7] Hassa PO, Haenni SS, Elser M, Hottiger MO (2006). Nuclear ADP-ribosylation reactions in mammalian cells: where are we today and where are we going. Microbiol Mol Biol Rev.

[8] Hassa PO, Hottiger MO (2008). The diverse biological roles of mammalian PARPS, a small but powerful family of poly-ADP-ribose polymerases. Front Biosci.

[9] D'Amours D, Desnoyers S, D'Silva I, Poirier GG (1999). Poly(ADP-ribosyl)ation reactions in the regulation of nuclear functions. Biochem J.

[10] Kim MY, Zhang T, Kraus WL (2005). Poly(ADP-ribosyl)ation by PARP-1: 'PAR-laying' NAD+ into a nuclear signal. Genes Dev.

[11] Ziegler M (2000). New functions of a long-known molecule. Emerging roles of NAD in cellular signaling. Eur J Biochem.

[12] Magni G, Amici A, Emanuelli M, Orsomando G, Raffaelli N, Ruggieri S (2004). Enzymology of NAD+ homeostasis in man. Cell Mol Life Sci.

[13] Berger F, Ramirez-Hernandez MH, Ziegler M (2004). The new life of a centenarian: signalling functions of NAD(P). Trends Biochem Sci.

[14] Hassa PO, Hottiger MO (2002). The functional role of poly(ADP-ribose)polymerase 1 as novel coactivator of NF-kappaB in inflammatory disorders. Cell Mol Life Sci.

[15] Alvarez-Gonzalez R, Althaus FR (1989). Poly(ADP-ribose) catabolism in mammalian cells exposed to DNA-damaging agents. Mutat Res.

[16] Oka J, Ueda K, Hayaishi O, Komura H, Nakanishi K (1984). ADP-ribosyl protein lyase. Purification, properties, and identification of the product. J Biol Chem.

[17] Ueda K, Oka J, Naruniya S, Miyakawa N, Hayaishi O (1972). Poly ADP-ribose glycohydrolase from rat liver nuclei, a novel enzyme degrading the polymer. Biochem Biophys Res Commun.

[18] Beneke S, Diefenbach J, Burkle A (2004). Poly(ADP-ribosyl)ation inhibitors: promising drug candidates for a wide variety of pathophysiologic conditions. Int J Cancer.

[19] Hassa PO (2009). The molecular "Jekyll and Hyde" duality of PARP1 in cell death and cell survival. Front Biosci.

[20] Grube K, Burkle A (1992). Poly(ADP-ribose) polymerase activity in mononuclear leukocytes of 13 mammalian species correlates with species-specific life span. Proc Natl Acad Sci U S A.

[21] Beneke S, Alvarez-Gonzalez R, Burkle A (2000). Comparative characterisation of poly(ADP-ribose) polymerase-1 from two mammalian species with different life span. Exp Gerontol.

[22] Althaus FR, Richter C (1987). ADP-ribosylation of proteins. Enzymology and biological significance. Mol Biol Biochem Biophys.

[23] Oei SL, Griesenbeck J, Schweiger M (1997). The role of poly(ADP-ribosyl)ation. Rev Physiol Biochem Pharmacol.

[24] Griesenbeck J, Oei SL, Mayer-Kuckuk P, Ziegler M, Buchlow G, Schweiger M (1997). Protein-protein interaction of the human poly(ADP-ribosyl)transferase depends on the functional state of the enzyme. Biochemistry.

[25] Potaman VN, Shlyakhtenko LS, Oussatcheva EA, Lyubchenko YL, Soldatenkov VA (2005). Specific binding of poly(ADP-ribose) polymerase-1 to cruciform hairpins. J Mol Biol.

[26] Till S, Ladurner AG (2009). Sensing NAD metabolites through macro domains. Front Biosci.

[27] Berger NA (1985). Poly(ADP-ribose) in the cellular response to DNA damage. Radiat Res.

[28] Berger NA, Sims JL, Catino DM, Berger SJ (1983). Poly(ADP-ribose) polymerase mediates the suicide response to massive DNA damage: studies in normal and DNA-repair defective cells. Princess Takamatsu Symp.

[29] Horton JK, Stefanick DF, Wilson SH (2005). Involvement of poly(ADP-ribose) polymerase activity in regulating Chk1-dependent apoptotic cell death. DNA Repair (Amst).

[30] Filipovic DM, Meng X, Reeves WB (1999). Inhibition of PARP prevents oxidant-induced necrosis but not apoptosis in LLC-PK1 cells. Am J Physiol.

[31] Ha HC, Snyder SH (1999). Poly(ADP-ribose) polymerase is a mediator of necrotic cell death by ATP depletion. Proc Natl Acad Sci U S A.

[32] Zong WX, Ditsworth D, Bauer DE, Wang ZQ, Thompson CB (2004). Alkylating DNA damage stimulates a regulated form of necrotic cell death. Genes Dev.

[33] Zong WX, Thompson CB (2006). Necrotic death as a cell fate. Genes Dev.

[34] Goto S, Xue R, Sugo N, Sawada M, Blizzard KK, Poitras MF, Johns DC, Dawson TM, Dawson VL, Crain BJ, Traystman RJ, Mori S, Hurn PD (2002). Poly(ADP-ribose) polymerase impairs early and long-term experimental stroke recovery. Stroke.

[35] Wang Y, Dawson VL, Dawson TM (2009). Poly(ADP-ribose) signals to mitochondrial AIF: A key event in parthanatos. Exp Neurol.

[36] Virag L, Szabo C (2002). The therapeutic potential of poly(ADP-ribose) polymerase inhibitors. Pharmacol Rev.

[37] Sarkar D, Fisher PB (2006). Molecular mechanisms of aging-associated inflammation. Cancer Lett.

[38] Harman D (1956). Aging: a theory based on free radical and radiation chemistry. J Gerontol.

[39] Nestelbacher R, Laun P, Vondrakova D, Pichova A, Schuller C, Breitenbach M (2000). The influence of oxygen toxicity on yeast mother cell-specific aging. Exp Gerontol.

[40] Wawryn J, Krzepilko A, Myszka A, Bilinski T (1999). Deficiency in superoxide dismutases shortens life span of yeast cells. Acta Biochim Pol.

[41] Unlu ES, Koc A (2007). Effects of deleting mitochondrial antioxidant genes on life span. Ann N Y Acad Sci.

[42] Kaeberlein M, Kirkland KT, Fields S, Kennedy BK (2005). Genes determining yeast replicative life span in a long-lived genetic background. Mech Ageing Dev.

[43] Hari R, Burde V, Arking R (1998). Immunological confirmation of elevated levels of CuZn superoxide dismutase protein in an artificially selected long-lived strain of Drosophila melanogaster. Exp Gerontol.

[44] Sohal RS, Weindruch R (1996). Oxidative stress, caloric restriction, and aging. Science.

[45] Blander G, de Oliveira RM, Conboy CM, Haigis M, Guarente L (2003). Superoxide dismutase 1 knock-down induces senescence in human fibroblasts. J Biol Chem.

[46] Kamata H, Hirata H (1999). Redox regulation of cellular signalling. Cell Signal.

[47] Chen Q, Fischer A, Reagan JD, Yan LJ, Ames BN (1995). Oxidative DNA damage and senescence of human diploid fibroblast cells. Proc Natl Acad Sci U S A.

[48] Esposito E, Cuzzocrea S (2009). Superoxide, NO, peroxynitrite and PARP in circulatory shock and inflammation. Front Biosci.

[49] Csiszar A, Wang M, Lakatta EG, Ungvari Z (2008). Inflammation and endothelial dysfunction during aging: role of NF-kappaB. J Appl Physiol.

[50] Gagne JP, Moreel X, Gagne P, Labelle Y, Droit A, Chevalier-Pare M, Bourassa S, McDonald D, Hendzel MJ, Prigent C, Poirier GG (2009). Proteomic Investigation of Phosphorylation Sites in Poly(ADP-ribose) Polymerase-1 and Poly(ADP-ribose) Glycohydrolase. J Proteome Res.

[51] Kauppinen TM, Chan WY, Suh SW, Wiggins AK, Huang EJ, Swanson RA (2006). Direct phosphorylation and regulation of poly(ADP-ribose) polymerase-1 by extracellular signal-regulated kinases 1/2. Proc Natl Acad Sci U S A.

[52] Cohen-Armon M, Visochek L, Rozensal D, Kalal A, Geistrikh I, Klein R, Bendetz-Nezer S, Yao Z, Seger R (2007). DNA-independent PARP-1 activation by phosphorylated ERK2 increases Elk1 activity: a link to histone acetylation. Mol Cell.

[53] Szabo C, Pacher P, Swanson RA (2006). Novel modulators of poly(ADP-ribose) polymerase. Trends Pharmacol Sci.

[54] Pollak N, Dolle C, Ziegler M (2007). The power to reduce: pyridine nucleotides--small molecules with a multitude of functions. Biochem J.

[55] Lau C, Niere M, Ziegler M (2009). The NMN/NaMN adenylyl-transferase (NMNAT) protein family. Front Biosci.

[56] Hilz H, Wielckens K, Adamietz P, Bredehorst R, Kreymeier A (1983). Functional aspects of mono- and poly(ADP-ribosyl)ation: subcellular distribution and ADP-ribosyl turnover under conditions of repair and 'starvation'. Princess Takamatsu Symp.

[57] Shall S (1983). ADP-ribosylation, DNA repair, cell differentiation and cancer. Princess Takamatsu Symp.

[58] Berger F, Lau C, Ziegler M (2007). Regulation of poly(ADP-ribose) polymerase 1 activity by the phosphorylation state of the nuclear NAD biosynthetic enzyme NMN adenylyl transferase 1. Proc Natl Acad Sci U S A.

[59] Guarente L, Picard F (2005). Calorie restriction--the SIR2 connection. Cell.

[60] Mair W, Dillin A (2008). Aging and survival: the genetics of life span extension by dietary restriction. Annu Rev Biochem.

[61] Mair W, Goymer P, Pletcher SD, Partridge L (2003). Demography of dietary restriction and death in Drosophila. Science.

[62] Weindruch R, Walford RL (1982). Dietary restriction in mice beginning at 1 year of age: effect on life-span and spontaneous cancer incidence. Science.

[63] Dhahbi JM, Kim HJ, Mote PL, Beaver RJ, Spindler SR (2004). Temporal linkage between the phenotypic and genomic responses to caloric restriction. Proc Natl Acad Sci U S A.

[64] Lin SJ, Kaeberlein M, Andalis AA, Sturtz LA, Defossez PA, Culotta VC, Fink GR, Guarente L (2002). Calorie restriction extends Saccharomyces cerevisiae lifespan by increasing respiration. Nature.

[65] Saunders LR, Verdin E (2007). Sirtuins: critical regulators at the crossroads between cancer and aging. Oncogene.

[66] Belenky P, Bogan KL, Brenner C (2007). NAD+ metabolism in health and disease. Trends Biochem Sci.

[67] Oberdoerffer P, Michan S, McVay M, Mostoslavsky R, Vann J, Park SK, Hartlerode A, Stegmuller J, Hafner A, Loerch P, Wright SM, Mills KD, Bonni A, Yankner BA, Scully R, Prolla TA, Alt FW, Sinclair DA (2008). SIRT1 redistribution on chromatin promotes genomic stability but alters gene expression during aging. Cell.

[68] Szabo C, Dawson VL (1998). Role of poly(ADP-ribose) synthetase in inflammation and ischaemia-reperfusion. Trends Pharmacol Sci.

[69] Hageman GJ, Stierum RH (2001). Niacin, poly(ADP-ribose) polymerase-1 and genomic stability. Mutat Res.

[70] Bitterman KJ, Anderson RM, Cohen HY, Latorre-Esteves M, Sinclair DA (2002). Inhibition of silencing and accelerated aging by nicotinamide, a putative negative regulator of yeast sir2 and human SIRT1. J Biol Chem.

[71] Altmeyer M, Messner S, Hassa PO, Fey M, Hottiger MO (2009). Molecular mechanism of poly(ADP-ribosyl)ation by PARP1 and identification of lysine residues as ADP-ribose acceptor sites. Nucleic Acids Res.

[72] Hassa PO, Haenni SS, Buerki C, Meier NI, Lane WS, Owen H, Gersbach M, Imhof R, Hottiger MO (2005). Acetylation of poly(ADP-ribose) polymerase-1 by p300/CREB-binding protein regulates coactivation of NF-kappaB-dependent transcription. J Biol Chem.

[73] Haenni SS, Hassa PO, Altmeyer M, Fey M, Imhof R, Hottiger MO (2008). Identification of lysines 36 and 37 of PARP-2 as targets for acetylation and auto-ADP-ribosylation. Int J Biochem Cell Biol.

[74] Chung HY, Sung B, Jung KJ, Zou Y, Yu BP (2006). The molecular inflammatory process in aging. Antioxid Redox Signal.

[75] Brooks CL, Gu W (2009). How does SIRT1 affect metabolism, senescence and cancer. Nat Rev Cancer.

[76] Rongvaux A, Shea RJ, Mulks MH, Gigot D, Urbain J, Leo O, Andris F (2002). Pre-B-cell colony-enhancing factor, whose expression is up-regulated in activated lymphocytes, is a nicotinamide phosphoribosyltransferase, a cytosolic enzyme involved in NAD biosynthesis. Eur J Immunol.

[77] Jia SH, Li Y, Parodo J, Kapus A, Fan L, Rotstein OD, Marshall JC (2004). Pre-B cell colony-enhancing factor inhibits neutrophil apoptosis in experimental inflammation and clinical sepsis. J Clin Invest.

[78] Ye SQ, Simon BA, Maloney JP, Zambelli-Weiner A, Gao L, Grant A, Easley RB, McVerry BJ, Tuder RM, Standiford T, Brower RG, Barnes KC, Garcia JG (2005). Pre-B-cell colony-enhancing factor as a potential novel biomarker in acute lung injury. Am J Respir Crit Care Med.

[79] Busso N, Karababa M, Nobile M, Rolaz A, Van Gool F, Galli M, Leo O, So A, De Smedt T (2008). Pharmacological inhibition of nicotinamide phosphoribosyltransferase/visfatin enzymatic activity identifies a new inflammatory pathway linked to NAD. PLoS ONE.

[80] Fukuzawa M, Satoh J, Muto G, Muto Y, Nishimura S, Miyaguchi S, Qiang XL, Toyota T (1997). Inhibitory effect of nicotinamide on in vitro and in vivo production of tumor necrosis factor-alpha. Immunol Lett.

[81] Ungerstedt JS, Blomback M, Soderstrom T (2003). Nicotinamide is a potent inhibitor of proinflammatory cytokines. Clin Exp Immunol.

[82] Cuzzocrea S (2005). Shock, inflammation and PARP. Pharmacol Res.

[83] Liu D, Pitta M, Mattson MP (2008). Preventing NAD(+) depletion protects neurons against excitotoxicity: bioenergetic effects of mild mitochondrial uncoupling and caloric restriction. Ann N Y Acad Sci.

[84] Liu D, Gharavi R, Pitta M, Gleichmann M, Mattson MP (2009). Nicotinamide prevents NAD+ depletion and protects neurons against excitotoxicity and cerebral ischemia: NAD+ consumption by SIRT1 may endanger energetically compromised neurons. Neuromolecular Med.

[85] Lim CS, Potts M, Helm RF (2006). Nicotinamide extends the replicative life span of primary human cells. Mech Ageing Dev.

[86] Van Gool F, Galli M, Gueydan C, Kruys V, Prevot PP, Bedalov A, Mostoslavsky R, Alt FW, De Smedt T, Leo O (2009). Intracellular NAD levels regulate tumor necrosis factor protein synthesis in a sirtuin-dependent manner. Nat Med.

[87] Yeung F, Hoberg JE, Ramsey CS, Keller MD, Jones DR, Frye RA, Mayo MW (2004). Modulation of NF-kappaB-dependent transcription and cell survival by the SIRT1 deacetylase. EMBO J.

[88] Kawahara TL, Michishita E, Adler AS, Damian M, Berber E, Lin M, McCord RA, Ongaigui KC, Boxer LD, Chang HY, Chua KF (2009). SIRT6 links histone H3 lysine 9 deacetylation to NF-kappaB-dependent gene expression and organismal life span. Cell.

[89] Lio D, Scola L, Romano GC, Candore G, Caruso C (2006). Immunological and immunogenetic markers in sporadic Alzheimer's disease. Aging Clin Exp Res.

[90] Pawelec G, Remarque E, Barnett Y, Solana R (1998). T cells and aging. Front Biosci.

[91] Haddad JJ (2003). Science review: redox and oxygen-sensitive trans-cripttion factors in the regulation of oxidant-mediated lung injury: role for hypoxia-inducible factor-1alpha. Crit Care.

[92] Kabe Y, Ando K, Hirao S, Yoshida M, Handa H (2005). Redox regulation of NF-kappaB activation: distinct redox regulation between the cytoplasm and the nucleus. Antioxid Redox Signal.

[93] Ghosh S, Hayden MS (2008). New regulators of NF-kappaB in inflammation. Nat Rev Immunol.

[94] Chung HY, Cesari M, Anton S, Marzetti E, Giovannini S, Seo AY, Carter C, Yu BP, Leeuwenburgh C (2009). Molecular inflammation: underpinnings of aging and age-related diseases. Ageing Res Rev.

[95] Kriete A, Mayo KL (2009). Atypical pathways of NF-kappaB activation and aging. Exp Gerontol.

[96] Karin M (1998). The NF-kappa B activation pathway: its regulation and role in inflammation and cell survival. Cancer J Sci Am.

[97] Ghosh S, May MJ, Kopp EB (1998). NF-kappa B and Rel proteins: evolutionarily conserved mediators of immune responses. Annu Rev Immunol.

[98] Perkins ND (2000). The Rel/NF-kappa B family: friend and foe. Trends Biochem Sci.

[99] Hoffmann A, Natoli G, Ghosh G (2006). Transcriptional regulation via the NF-kappaB signaling module. Oncogene.

[100] Silverman N, Maniatis T (2001). NF-kappaB signaling pathways in mammalian and insect innate immunity. Genes Dev.

[101] Bonizzi G, Karin M (2004). The two NF-kappaB activation pathways and their role in innate and adaptive immunity. Trends Immunol.

[102] Gerondakis S, Grumont R, Gugasyan R, Wong L, Isomura I, Ho W, Banerjee A (2006). Unravelling the complexities of the NF-kappaB signalling pathway using mouse knockout and transgenic models. Oncogene.

[103] Kim HJ, Yu BP, Chung HY (2002). Molecular exploration of age-related NF-kappaB/IKK downregulation by calorie restriction in rat kidney. Free Radic Biol Med.

[104] Adler AS, Sinha S, Kawahara TL, Zhang JY, Segal E, Chang HY (2007). Motif module map reveals enforcement of aging by continual NF-kappaB activity. Genes Dev.

[105] Hayden MS, Ghosh S (2008). Shared principles in NF-kappaB signaling. Cell.

[106] Hayden MS, Ghosh S (2004). Signaling to NF-kappaB. Genes Dev.

[107] Chambers SM, Shaw CA, Gatza C, Fisk CJ, Donehower LA, Goodell MA (2007). Aging hematopoietic stem cells decline in function and exhibit epigenetic dysregulation. PLoS Biol.

[108] Adler AS, Kawahara TL, Segal E, Chang HY (2008). Reversal of aging by NFkappaB blockade. Cell Cycle.

[109] Donato AJ, Black AD, Jablonski KL, Gano LB, Seals DR (2008). Aging is associated with greater nuclear NFkappaB, reduced IkappaBalpha, and increased expression of proinflammatory cytokines in vascular endothelial cells of healthy humans. Aging Cell.

[110] Kim DH, Kim JY, Yu BP, Chung HY (2008). The activation of NF-kappaB through Akt-induced FOXO1 phosphorylation during aging and its modulation by calorie restriction. Biogerontology.

[111] Ungvari Z, Orosz Z, Labinskyy N, Rivera A, Xiangmin Z, Smith K, Csiszar A (2007). Increased mitochondrial H2O2 production promotes endothelial NF-kappaB activation in aged rat arteries. Am J Physiol Heart Circ Physiol.

[112] Wang ZQ, Auer B, Stingl L, Berghammer H, Haidacher D, Schweiger M, Wagner EF (1995). Mice lacking ADPRT and poly(ADP-ribosyl)ation develop normally but are susceptible to skin disease. Genes Dev.

[113] Shall S, de Murcia G (2000). Poly(ADP-ribose) polymerase-1: what have we learned from the deficient mouse model. Mutat Res.

[114] Burkart V, Wang ZQ, Radons J, Heller B, Herceg Z, Stingl L, Wagner EF, Kolb H (1999). Mice lacking the poly(ADP-ribose) polymerase gene are resistant to pancreatic beta-cell destruction and diabetes development induced by streptozocin. Nat Med.

[115] Szabo C, Cuzzocrea S, Zingarelli B, O'Connor M, Salzman AL (1997). Endothelial dysfunction in a rat model of endotoxic shock. Importance of the activation of poly (ADP-ribose) synthetase by peroxynitrite. J Clin Invest.

[116] Oliver FJ, Menissier-de Murcia J, Nacci C, Decker P, Andriantsitohaina R, Muller S, de la Rubia G, Stoclet JC, de Murcia G (1999). Resistance to endotoxic shock as a consequence of defective NF-kappaB activation in poly (ADP-ribose) polymerase-1 deficient mice. EMBO J.

[117] Ullrich O, Diestel A, Eyupoglu IY, Nitsch R (2001). Regulation of microglial expression of integrins by poly(ADP-ribose) polymerase-1. Nat Cell Biol.

[118] Tulin A, Chinenov Y, Spradling A (2003). Regulation of chromatin structure and gene activity by poly(ADP-ribose) polymerases. Curr Top Dev Biol.

[119] Jagtap P, Szabo C (2005). Poly(ADP-ribose) polymerase and the therapeutic effects of its inhibitors. Nat Rev Drug Discov.

[120] Ruf A, Mennissier de Murcia J, de Murcia G, Schulz GE (1996). Structure of the catalytic fragment of poly(AD-ribose) polymerase from chicken. Proc Natl Acad Sci U S A.

[121] Ruf A, de Murcia G, Schulz GE (1998). Inhibitor and NAD+ binding to poly(ADP-ribose) polymerase as derived from crystal structures and homology modeling. Biochemistry.

[122] Oliver AW, Ame JC, Roe SM, Good V, de Murcia G, Pearl LH (2004). Crystal structure of the catalytic fragment of murine poly(ADP-ribose) polymerase-2. Nucleic Acids Res.

[123] Vaziri H, West MD, Allsopp RC, Davison TS, Wu YS, Arrowsmith CH, Poirier GG, Benchimol S (1997). ATM-dependent telomere loss in aging human diploid fibroblasts and DNA damage lead to the post-translational activation of p53 protein involving poly(ADP-ribose) polymerase. EMBO J.

[124] Cuzzocrea S, McDonald MC, Mazzon E, Dugo L, Serraino I, Threadgill M, Caputi AP, Thiemermann C (2002). Effects of 5-aminoisoquinolinone, a water-soluble, potent inhibitor of the activity of poly (ADP-ribose) polymerase, in a rodent model of lung injury. Biochem Pharmacol.

[125] Kiefmann R, Heckel K, Dorger M, Schenkat S, Stoeckelhuber M, Wesierska-Gadek J, Goetz AE (2003). Role of poly(ADP-ribose) synthetase in pulmonary leukocyte recruitment. Am J Physiol Lung Cell Mol Physiol.

[126] Szabo C, Lim LH, Cuzzocrea S, Getting SJ, Zingarelli B, Flower RJ, Salzman AL, Perretti M (1997). Inhibition of poly (ADP-ribose) synthetase attenuates neutrophil recruitment and exerts antiinflammatory effects. J Exp Med.

[127] Kroger H, Miesel R, Dietrich A, Ohde M, Rajnavolgyi E, Ockenfels H (1996). Synergistic effects of thalidomide and poly (ADP-ribose) polymerase inhibition on type II collagen-induced arthritis in mice. Inflammation.

[128] Szabo C, Virag L, Cuzzocrea S, Scott GS, Hake P, O'Connor MP, Zingarelli B, Salzman A, Kun E (1998). Protection against peroxynitrite-induced fibroblast injury and arthritis development by inhibition of poly(ADP-ribose) synthase. Proc Natl Acad Sci U S A.

[129] Mabley JG, Jagtap P, Perretti M, Getting SJ, Salzman AL, Virag L, Szabo E, Soriano FG, Liaudet L, Abdelkarim GE, Hasko G, Marton A, Southan GJ, Szabo C (2001). Anti-inflammatory effects of a novel, potent inhibitor of poly (ADP-ribose) polymerase. Inflamm Res.

[130] Mazzon E, Serraino I, Li JH, Dugo L, Caputi AP, Zhang J, Cuzzocrea S (2001). GPI 6150, a poly (ADP-ribose) polymerase inhibitor, exhibits an anti-inflammatory effect in rat models of inflammation. Eur J Pharmacol.

[131] von Lukowicz T, Hassa PO, Lohmann C, Boren J, Braunersreuther V, Mach F, Odermatt B, Gersbach M, Camici GG, Stahli BE, Tanner FC, Hottiger MO, Luscher TF, Matter CM (2008). PARP1 is required for adhesion molecule expression in atherogenesis. Cardiovasc Res.

[132] Garcia Soriano F, Virag L, Jagtap P, Szabo E, Mabley JG, Liaudet L, Marton A, Hoyt DG, Murthy KG, Salzman AL, Southan GJ, Szabo C (2001). Diabetic endothelial dysfunction: the role of poly(ADP-ribose) polymerase activation. Nat Med.

[133] Radovits T, Seres L, Gero D, Berger I, Szabo C, Karck M, Szabo G (2007). Single dose treatment with PARP-inhibitor INO-1001 improves aging-associated cardiac and vascular dysfunction. Exp Gerontol.

[134] Zingarelli B, Hake PW, O'Connor M, Denenberg A, Wong HR, Kong S, Aronow BJ (2004). Differential regulation of activator protein-1 and heat shock factor-1 in myocardial ischemia and reperfusion injury: role of poly(ADP-ribose) polymerase-1. Am J Physiol Heart Circ Physiol.

[135] Zingarelli B, Salzman AL, Szabo C (1998). Genetic disruption of poly (ADP-ribose) synthetase inhibits the expression of P-selectin and intercellular adhesion molecule-1 in myocardial ischemia/reperfusion injury. Circ Res.

[136] Ha HC, Hester LD, Snyder SH (2002). Poly(ADP-ribose) polymerase-1 dependence of stress-induced transcription factors and associated gene expression in glia. Proc Natl Acad Sci U S A.

[137] Hassa PO, Covic M, Hasan S, Imhof R, Hottiger MO (2001). The enzymatic and DNA binding activity of PARP-1 are not required for NF-kappa B coactivator function. J Biol Chem.

[138] Hassa PO, Buerki C, Lombardi C, Imhof R, Hottiger MO (2003). Transcriptional coactivation of nuclear factor-kappaB-dependent gene expression by p300 is regulated by poly(ADP)-ribose polymerase-1. J Biol Chem.

[139] Hauschildt S, Scheipers P, Bessler W, Schwarz K, Ullmer A, Flad HD, Heine H (1997). Role of ADP-ribosylation in activated monocytes/macrophages. Adv Exp Med Biol.

[140] Soriano FG, Pacher P, Mabley J, Liaudet L, Szabo C (2001). Rapid reversal of the diabetic endothelial dysfunction by pharmacological inhibition of poly(ADP-ribose) polymerase. Circ Res.

[141] Koch-Nolte F, Haag F (1997). Mono(ADP-ribosyl)transferases and related enzymes in animal tissues: Emerging gene families. Adv Exp Med Biol.

[142] Yelamos J, Monreal Y, Saenz L, Aguado E, Schreiber V, Mota R, Fuente T, Minguela A, Parrilla P, de Murcia G, Almarza E, Aparicio P, Menissier-de Murcia J (2006). PARP-2 deficiency affects the survival of CD4+CD8+ double-positive thymocytes. EMBO J.

[143] Kofler J, Otsuka T, Zhang Z, Noppens R, Grafe MR, Koh DW, Dawson VL, de Murcia JM, Hurn PD, Traystman RJ (2006). Differential effect of PARP-2 deletion on brain injury after focal and global cerebral ischemia. J Cereb Blood Flow Metab.

[144] Popoff I, Jijon H, Monia B, Tavernini M, Ma M, McKay R, Madsen K (2002). Antisense oligonucleotides to poly(ADP-ribose) polymerase-2 ameliorate colitis in interleukin-10-deficient mice. J Pharmacol Exp Ther.

[145] Veres B, Gallyas F Jr, Varbiro G, Berente Z, Osz E, Szekeres G, Szabo C, Sumegi B (2003). Decrease of the inflammatory response and induction of the Akt/protein kinase B pathway by poly-(ADP-ribose) polymerase 1 inhibitor in endotoxin-induced septic shock. Biochem Pharmacol.

[146] Sakitani K, Nishizawa M, Inoue K, Masu Y, Okumura T, Ito S (1998). Synergistic regulation of inducible nitric oxide synthase gene by CCAAT/enhancer-binding protein beta and nuclear factor-kappaB in hepatocytes. Genes Cells.

[147] Catron KM, Brickwood JR, Shang C, Li Y, Shannon MF, Parks TP (1998). Cooperative binding and synergistic activation by RelA and C/EBPbeta on the intercellular adhesion molecule-1 promoter. Cell Growth Differ.

